# Methodological and Statistical Comments on CASPIAN-III Study

**DOI:** 10.5812/hepatmon.10161

**Published:** 2013-05-25

**Authors:** Ali Kabir

**Affiliations:** 1 Department of Epidemiology, Faculty of Public Health, Shahid Beheshti University of Medical Sciences, Tehran, IR Iran

**Keywords:** Cluster Analysis, Linear Models, Cohort Effect

*Dear Editor,*

Kelishadi and her colleagues have done a great job for determining students’ high risk behaviors (1) and have published part of this study as percentiles of aminotransaminases in Iranian children (2) These data are only from Iran, a country in Middle East and not children of Middle East and North Africa (MENA). Only children between 10 and 18 years are studied and not all children like younger than 10 years. Moreover, only 27 provinces from 31 provinces have been selected and not even all provinces of Iran. Data are really valuable but not representative of MENA. These are not even completely generalizable to Iranian children because neither covers all provinces nor all ages. Despite I did not see any calculation based sample size estimation neither in this paper (2) nor in their previous paper about the methodology of this study (1); but, sample size seems relatively sufficient. However, sampling method is more important than sample size for generalizability. Authors claimed that difference in their findings with previous study in Tehran, which has revealed lower limit for upper normal limits for ALT and AST (3), can be due to sampling, which is only from one city and not whole country and this difference may be also due to lower sample size. They have mentioned that the population studied in Tehran was less than one-fourth of the current nationwide study. It should be considered this "one-forth", accounted for 975 samples from one province. It is higher than your sample from Tehran and more homogenous than your nationwide sample. You should not compare your total sample size with the number of studied people in one province. Their results seems more representative to Tehran due to your mentioned limitations specifically when Poustchi et al. (3) have excluded subjects having abnormal values for factors that correlated with ALT in their study. In different parts of the paper (2), authors have expressed that there are linear associations. Why the authors believe that this association is linear? Is there any pre-specified hypothesis? Most of the time, the associations are not linear specifically when there is no significant linear relationship or the linear association is weak. In this study R-squares are low and the shape of the association between BMI and age with AST and ALT (Figures 1 and 2) shows that this association is not linear. It is not offered to fit a line for such association. P values are useless when the sample size is large even they become significant. R-squares are so weak and near to zero in most of these linear regressions which shows there is not a linear association. Even with such large sample size, P values were not significant for linear regression in some instances like WHtR and AST as you have mentioned. So, these associations are not linear in your study. In addition, cluster analysis is needed when cluster sampling is done. This analysis will specifically affect on all analytical results (like significancy and strength of correlations and regressions) and confidence intervals of all descriptive statistics. But, authors did not consider such analysis in their results. Kelishadi et al (2) presented the association between age and AST or ALT. I would like to reinforce that it is a cross sectional study which only shows children with higher age have lower level of ALT and AST except for ALT in females. It is not a cohort showing that with increasing age the ALT or AST level increases. If we had followed a cohort of children for some years and observe that ALT or AST are increasing with age in the same persons, then we could conclude such sentence. These are different concepts which usually misunderstood. It is known as age effect and cohort effect (4).
